# Weight loss, malnutrition, and cachexia in COVID‐19: facts and numbers

**DOI:** 10.1002/jcsm.12674

**Published:** 2020-12-31

**Authors:** Markus S. Anker, Ulf Landmesser, Stephan von Haehling, Javed Butler, Andrew J.S. Coats, Stefan D. Anker

**Affiliations:** ^1^ Department of Cardiology Universitätsmedizin Berlin, Charité—Campus Benjamin‐Franklin Hindenburgdamm 30 12200 Berlin Germany; ^2^ Berlin Institute of Health Center for Regenerative Therapies (BCRT) Berlin Germany; ^3^ German Centre for Cardiovascular Research (DZHK), partner site Berlin Berlin Germany; ^4^ Berlin Institute of Health (BIH) Berlin Germany; ^5^ Department of Cardiology and Pneumology University of Göttingen Medical Center Göttingen Germany; ^6^ German Centre for Cardiovascular Research (DZHK), partner site Göttingen Göttingen Germany; ^7^ Department of Medicine University of Mississippi Jackson MS USA; ^8^ University of Warwick Warwick UK; ^9^ Department of Cardiology Charité—Universitätsmedizin Berlin, Campus Virchow Klinikum Berlin Germany

**Keywords:** COVID‐19, Cachexia, Weight loss, Malnutrition, Epidemiology

## Abstract

Patients with COVID‐19 disease are prone to develop significant weight loss and clinical cachexia. Three reports with altogether 589 patients that reported on weight loss and cachexia in COVID‐19 were identified. Disease severity of patients and the timing of the assessment during the disease course in these patients were variable—65 patients (11%) were intensive care treated at the time of assessment, and 183 (31%) were cared for in sub‐intensive or intermediate care structures. The frequency of weight loss ≥5% (that defines cachexia) was 37% (range 29–52%). Correlates of weight loss occurrence were reported to be raised C‐reactive protein levels, impaired renal function status, and longer duration of COVID‐19 disease. Underweight status by WHO criteria (BMI < 18.5 kg/m^2^) was only observed in 4% of patients analysing data from seven studies with 6661 patients. Cachexia assessment in COVID‐19 needs assessment of weight loss. COVID‐19 associated cachexia is understood to affect muscle and fat tissue as is also seen in many other chronic illness‐associated forms of cachexia. There are many factors that can contribute to body wasting in COVID‐19, and they include loss of appetite and taste, fever and inflammation, immobilization, as well as general malnutrition, catabolic–anabolic imbalance, endocrine dysfunction, and organ‐specific complications of COVID‐19 disease such as cardiac and renal dysfunction. Treatment of COVID‐19 patients should include a focus on nutritional support and rehabilitative exercise whenever possible. Specific anti‐cachectic therapies for COVID‐19 do not exist, but constitute a high medical need to prevent long‐term disability due to acute COVID‐19 disease.

## Introduction

Fatigue and poor functional status are hallmarks of patients infected with severe acute respiratory syndrome coronavirus type 2 (SARS‐CoV‐2), of those being hospitalized for COVID‐19, and also of those recuperating (often over many months) from the acute illness. Muscle wasting and cachexia, and associated with these malnutrition as well as complex metabolic alterations, are considered a major reason for both acute clinical problems and longer term damage to the health of patients affected by post‐COVID‐19 chronic disease.^1^

## Weight loss during COVID‐19

Weight loss in COVID‐19 is caused by many different factors. First of all, the SARS‐CoV‐2 infection can cause major inflammation, particularly pronounced in patients with severe and critical COVID‐19 disease.^2^ Endothelial and epithelial cell death combined with vascular leakage can trigger production of chemokines and cytokines, and may result in a massive inflammatory reaction causing disturbed tissue homeostasis.^1^, ^3^, ^4^ Acute phase proteins such as: TNFα, ferritin, C‐reactive protein (CRP), fibroblast growth factor, IL‐factors, NF‐κB, interferon‐γ are produced manifold and circulated, causing further dysregulation of metabolism and proteolysis.^4^

Secondly, malnutrition is frequently seen in COVID‐19 patients.^5^, ^6^, ^7^ Disease‐associated reductions in food intake and malnutrition can contribute to tissue wasting. Also, appetite loss, ageusia (loss of taste), fever, and sedation further contribute to malnutrition and cachexia in COVID‐19. All of these problems can lead to catabolic overdrive (likely associated with anabolic failure) and hence trigger weight loss in COVID‐19.

Thirdly, immobilization can also significantly contribute to muscle wasting and sarcopenia in COVID‐19.^8^ Ultimately, it seems clear that the negative ‘synergy’ of all these factors together can cause significant body wasting in COVID‐19 patients as clinically often observed.^1^

## Malnutrition frequency in COVID‐19

Two studies, constituting 395 hospitalized COVID‐19 patients in total, studied malnutrition in COVID‐19 utilizing the Mini Nutritional Assessment score. They found that 42% of hospitalized patients with COVID‐19 were at risk of malnutrition and 28% of these patients were considered malnourished at the time of assessment.^5^, ^6^ Another study in 114 consecutive, hospitalized COVID‐19 patients found similar results using the Global Leadership Initiative on Malnutrition (GLIM) criteria with 42% (48/114 patients) being malnourished.^7^

## Cachexia diagnosis and frequency during COVID‐19

When weight loss reaches clinical significance, cachexia due to COVID‐19 can be diagnosed. A consensus definition that is specific for COVID‐19 patients does not yet exist. We suggest to apply the general consensus definition of cachexia^9^ also to this illness. This means that cachexia in COVID‐19 can be diagnosed when ≥5% weight loss is observed, functional status is impaired, and metabolic derangement (e.g. inflammation) can be documented.

Until 12 December 2020, we have identified three peer‐reviewed publications investigating the occurrence of weight loss before, during, or after hospital treatment of in total 589 COVID‐19 patients (Table 1). One study^10^ was performed at the time of hospital admission and reported on weight loss ≥5% before the index hospitalization, one study^11^ investigated weight loss ≥5% until the examination date during hospitalization, and one study^6^ assessed weight loss of patients in remission (i.e. after hospitalization). This clearly shows that weight loss assessment during COVID‐19 disease is not yet harmonized. We recommend that such harmonization is sought. This will support future investigations, making studies better comparable. This will also be a requirement if specific intervention trials were to be planned.

**Table 1:** Summary of data reported in published reports on weight loss and cachexia in COVID‐19 patients

Parameters	Pironi *et al*.^11^ (*n* = 268)	Di Filippo *et al*.^6^ (*n* = 213)	Allard *et al*.^10^ (*n* = 108)
Date published	August 2020	October 2020	November 2020
Origin of data	Bologna, Italy	Milan, Italy	Bobigny, France
Patient inclusion	Retrospective	Retrospective	Retrospective
Time of assessment	During treatment of COVID‐19 (in hospital)	After COVID‐19 in remission (outpatients)	At time of hospital admission for COVID‐19
Age (years), mean ± SD or median (IQR)	74 (63–84)	59 (50–68)	62 ± 16
Female, *n* (%)	121 (45)	71 (33)	44 (41)
BMI (kg/m^2^), mean ± SD or median (IQR)	25.1 (22.0–27.8)	27.1 (24.7–31.0)	28.8 ± 6.2
Number of hospitalized patients, *n* (%)	268 (100)	156 (73)^a^	108 (100)
Intensive care unit treatment, *n* (%)	46 (17)	5 (3)	14 (13)
Intermediate care unit treatment^b^	183 (68)	NA	NA
Severe COVID‐19^c^, *n* (%)	NA	NA	34 (32)
Mild/moderate COVID‐19, *n* (%)	NA	NA	74 (69)
Weight loss ≥5% during COVID‐19 or in the last month, *n* (%)	65 (52)^d^	61 (29)	40 (37)
Observed weight loss ≥10% in last 6 months, *n* (%)	NA	NA	10 (10)

^a^ Previously hospitalized patients.

^b^ Intermediate care unit treatment or care in ‘sub‐intensive care’ unit as described by authors.

^c^ Defined as nasal oxygen ≥6 L/min.

^d^ Data regarding weight loss were available in 125 of 268 patients.

Overall, weight loss ≥5% was reported in 37% of COIVD‐19 patients with available data (166/446 patients, Table 1). The study by Pironi *et al*.^11^ with most patients in intensive, sub‐intensive, and intermediate care units (≥85%) showed the highest incidence of weight loss ≥5% with 52% of patients (65/125 patients). Therefore, these patients seem to be in special need of nutritional attention and monitoring including enteral or parenteral nutrition.^12^ Also, COVID‐19 patients mainly treated in a normal COVID‐19 ward had a risk of weight loss ≥5% of about 33%.^6^, ^11^

In addition to the reports discussed in Table 1, the study of Essomba *et al*.^13^ reported on weight loss in 30 COVID‐19 patients based on self‐reported statements that such weight loss was present. Here, 9 of 30 patients (30%) confirmed the presence of weight loss. As there were no further details on this information, we did not add this study in Table 1.

We also investigated BMI reports in COVID‐19 studies, but this is in no way a complete analysis of available data. Nevertheless, only a minority of COVID‐19 clinical trials or observational studies report on the presence of underweight. Underweight (as per the WHO definition) with a BMI of <18.5 kg/m^2^ was seen in only 4% of COVID‐19 patients (267/6661 patients) (Table 2). We believe that the WHO classification of body weight^18^ is not useful to define clinical cachexia, as is also not the case in cancer,^19^, ^20^, ^21^ chronic kidney disease,^22^, ^23^, ^24^, ^25^ COPD,^25^, ^26^, ^27^ or heart failure.^28^, ^29^, ^30^

**Table 2:** Examples of key data reported in important published reports on the frequency of underweight clinical status (i.e. BMI < 18.5 kg/m^2^) in COVID‐19 patients

Parameters	Cai *et al*.^14^ (*n* = 383)	Pironi *et al*.^11^ (*n* = 268)	Jung *et al*.^15^ (n = 3788)	Zakeri *et al*.^16^ (*n* = 1572)	Di Filippo *et al*.^6^ (*n* = 213)	Allard *et al*.^10^ (*n* = 108)	Al‐Salameh *et al*.^17^ (*n* = 329)
Date published	July 2020	August 2020	August 2020	October 2020	October 2020	November 2020	November 2020
Age (years), mean ± SD or median (IQR)	NA^a^	74 (63–84)	54 ± 14	70 (56–82)	59 (50–68)	62 ± 16	NA^b^
Female, *n* (%)	200 (52)	121 (45)	2351 (62)	686 (4)	71 (33)	44 (41)	143 (43)
BMI (kg/m^2^), mean ± SD or median (IQR)	NA	25.1 (22.0–27.8)	24.0 ± 3.4	26.7 (22.7–31.9)	27.1 (24.7–31.0)	28.8 ± 6.2	NA
Underweight (BMI ≤ 18.5 kg/m^2^), *n* (%)	16 (4)	24 (9)	132 (3)	66 (4)	4 (2)	5 (5)^c^	20 (6)

^a^ Median age in sub‐groups ranged from 36–50 years.

^b^ Median age in sub groups ranged from 66–85 years.

^c^ Body mass index (BMI) ≤18.5 or ≤21 kg/m^2^, if age ≥70 years.

## Predictors of cachexia in COVID‐19

There are very limited data on the predictors of weight loss and cachexia in COVID‐19. We suggest that anything that predicts a severe course of COVID‐19 disease also predicts cachexia and muscle wasting. Weight loss in COVID‐19 patients has been found to positively correlate with higher CRP levels, reduced kidney function, and longer duration of disease.^6^ Possible associations likely also include abnormal markers of cardiac and kidney function in COVID‐19,^31^, ^32^ as cachexia in acute and in chronic COVID‐19 disease may also develop as a secondary consequence of cardiac and renal problems. Therapies that prevent cardiac and renal events may therefore also be useful in the short‐term and long‐term care of patients with COVID‐19 or after COVID‐19 acute illness. Such therapies SGLT2 inhibitors—one trial with dapagliflozin is running in at least 900 COVID‐19 patients (DARE‐19, started during hospitalization, duration of treatment 30 days, see clinicaltrials.gov: NCT 04350593^33^), but studies focusing on the post COVID‐19 disease process are lacking. COVID‐19 has a high potential to induce fibrosis in the lung,^34^ in the heart,^35^ and elsewhere in the body, which can also contribute to a chronic wasting processes. Because of this, we believe that therapy with mineralo‐corticoid receptor antagonists may also be useful in acute COVID‐19 and in the post COVID‐19 situation.

## Summary

Patients with COVID‐19 disease are prone to develop significant weight loss and clinical cachexia. Three reports with a total of 589 patients were identified that reported on weight loss and cachexia in COVID‐19. The frequency of weight loss ≥5% (that defines cachexia) was 37% (range 29–52%). Cachexia assessment in COVID‐19 needs assessment of weight loss. COVID‐19 associated cachexia is understood to affect muscle and fat tissue as is also seen in many other chronic illness‐associated cachexias. There are many metabolic and nutritional factors that can contribute to body wasting in COVID‐19, and they likely also include organ specific complication of COVID‐19 disease like cardiac and renal dysfunction. Treatment of COVID‐19 patients should focus on nutritional support and rehabilitative exercise whenever possible. Specific anti‐cachectic therapies for COVID‐19 do not exist, but constitute a high medical need to prevent long‐term disability due to acute COVID‐19 disease.

## Conflict of interest

M.S.A. reports personal fees from Servier, outside the submitted work.

U.L. reports personal lecture or advisory fees from Amgen, Novartis, Sanofi, Bayer, Abbott, Böhringer, Daichy Sankyo, and NovoNordisk.

S.v.H. reports received personal fees from AstraZeneca, Bayer, Boehringer Ingelheim, BRAHMS, Chugai, Grünenthal, Helsinn, Hexal, Novartis, Respicardia, Roche, Sorin, and Vifor. SvH reports research support from Amgen, AstraZeneca, Boehringer Ingelheim, IMI, and the German Center for Cardiovascular Research (DZHK), all outside the submitted work.

J.B. serves as a consultant for Abbott, Adrenomed, Amgen, Applied Therapeutics, Array, Astra Zeneca, Bayer, BerlinCures, Boehringer Ingelheim, CVRx, G3 Pharmaceutical, Impulse Dynamics, Innolife, Janssen, LivaNova, Luitpold, Medtronic, Merck, Novartis, NovoNordisk, Relypsa, Sequana Medical, V‐Wave Limited, and Vifor.

A.J.C. has received personal fees from Astra Zeneca, Bayer, Boehringer Ingelheim, Menarini, Novartis, Nutricia, Servier, Vifor, Abbott, Actimed, Arena, Cardiac Dimensions, Corvia, CVRx, Enopace, ESN Cleer, Faraday, WL Gore, Impulse Dynamics, and Respicardia, all outside the submitted work.

S.D.A. reports grants from Vifor Int and Abbott and personal fees from Vifor, Bayer, Boehringer Ingelheim, Novartis, Servier, Abbott, Actimed, Cardiac Dimensions, and Impulse Dynamics, all outside the submitted work.
